# The Dual Origin of the Yeast Mitochondrial Proteome

**DOI:** 10.1002/1097-0061(20000930)17:3<170::AID-YEA25>3.0.CO;2-V

**Published:** 2000

**Authors:** Olof Karlberg, Björn Canbäck, Charles G. Kurland, Siv G. E. Andersson

**Affiliations:** 1 Department of Molecular Evolution Uppsala University Uppsala 752 34 Sweden; 2 Department of Molecular Evolution Evolutionary Biology Center Norbyvägen 18C Uppsala 752 34 Sweden

## Abstract

We propose a scheme for the origin of mitochondria based on phylogenetic reconstructions with more than 400 yeast nuclear genes that encode mitochondrial proteins. Half of the yeast mitochondrial proteins have no discernable bacterial homologues, while one-tenth are unequivocally of α-proteobacterial origin. These data suggest that the majority of genes encoding yeast mitochondrial proteins are descendants of two different genomic lineages that have evolved in different modes. First, the ancestral free-living α-proteobacterium evolved into an endosymbiont of an anaerobic host. Most of the ancestral bacterial genes were lost, but a small fraction of genes supporting bioenergetic and translational processes were retained and eventually transferred to what became the host nuclear genome. In a second, parallel mode, a larger number of novel mitochondrial genes were recruited from the nuclear genome to complement the remaining genes from the bacterial ancestor. These eukaryotic genes, which are primarily involved in transport and regulatory functions, transformed the endosymbiont into an ATP-exporting organelle.


**Yeast**
*Yeast* 2000; **17**: 170–187.

**Research Article**

### The dual origin of the yeast mitochondrial proteome

Olof Karlberg, Björn Canbäck, Charles G. Kurland and Siv G. E. Andersson*
*Department of Molecular Evolution, Uppsala University, 751 24 Uppsala, Sweden*

*Correspondence to: S. G. E. Andersson, Department of Molecular Evolution, Evolutionary Biology Center, Norbyvägen 18C, 752 34 Uppsala, Sweden. E-mail: Siv.Andersson@ebc.uu.se

Received: 30 May 2000
Accepted: 14 July 2000

### Abstract

**We propose a scheme for the origin of mitochondria based on phylogenetic reconstructions with more than 400 yeast nuclear genes that encode mitochondrial proteins. Half of the yeast mitochondrial proteins have no discernable bacterial homologues, while one-tenth are unequivocally of $\alpha$-proteobacterial origin. These data suggest that the majority of genes encoding yeast mitochondrial proteins are descendants of two different genomic lineages that have evolved in different modes. First, the ancestral free-living $\alpha$-proteobacterium evolved into an endosymbiont of an anaerobic host. Most of the ancestral bacterial genes were lost, but a small fraction of genes supporting bioenergetic and translational processes were retained and eventually transferred to what became the host nuclear genome. In a second, parallel mode, a larger number of novel mitochondrial genes were recruited from the nuclear genome to complement the remaining genes from the bacterial ancestor. These eukaryotic genes, which are primarily involved in transport and regulatory functions, transformed the endosymbiont into an ATP-exporting organelle. Copyright © 2000 John Wiley & Sons, Ltd.**

**Keywords:** ***Saccharomyces cerevisiae*; *Rickettsia prowazekii*; mitochondria; comparative genomics; evolution**

### Introduction

The endosymbiotic theory for the origin of mitochondria implies in its simplest form that the mitochondrial proteins are encoded by genes that have descended from an ancestral $\alpha$-proteobacterium (Gray, 1992). We have previously compared the genome of the $\alpha$-proteobacterial parasite *Rickettsia prowazekii* to an ensemble of genes from mitochondrial genomes (Andersson *et al.*, 1998). We detect two striking patterns: First, the ATP generating pathway of *R. prowazekii* is identical to that of mitochondria: both systems are incapable of glycolysis. Likewise, both systems generate ATP by Krebs cycle oxidations coupled to a cytochrome-mediated electron transport system that terminates in cytochrome oxidase. Second, phylogenetic reconstructions based on rRNA, ribosomal proteins, heat shock proteins, NADH dehydrogenase subunits, cytochrome oxidase and cytochrome *b* reveal a close evolutionary relationship between mitochondria and aerobic $\alpha$-proteobacteria (Olsen *et al.*, 1994; Viale and Arakaki, 1994; Andersson *et al.*, 1998; Sicheritz-Ponten *et al.*, 1998; Gray *et al.*, 1999). The most parsimonious interpretation of these results is that both *Rickettsia* and mitochondria arose from an ancestral $\alpha$-proteobacterium that had the capacity for oxidative phosphorylation, in accordance with an important prediction of the endosymbiotic theory.

Proponents of different versions of the endosymbiotic theory have argued that there was a massive transfer of genes from the endosymbiont into the nuclear genome during the evolution of the mitochondrion (Martin and Muller, 1998; Gray *et al.*, 1999). Indeed, almost all of the genes encoding the proteins of modern mitochondria are found in the nuclear genomes of their host cells (Gray *et al.*, 1999). However, our initial comparisons of coding sequences from the *Rickettsia* genome with nuclear homologues encoding mitochondrial proteins in *Saccharomyces cerevisiae* provided indications of a more complex evolutionary scenario (Andersson *et al.*, 1998). On the one hand, roughly 40% of the

Copyright © 2000 John Wiley & Sons, Ltd.

genes encoding information transfer and energy production in the mitochondrial proteome have homologues in *Rickettsia*. On the other hand, many proteins in other functional classes lack homologues in *Rickettsia*. To examine in more detail the origin and evolution of these proteins, we have now carried out phylogenetic reconstructions based on a set of more than 400 mitochondrial proteins in *Saccharomyces cerevisiae*.

We find that more than half of the proteins of the yeast mitochondrial proteome have no counterparts in bacteria. Phylogenetic reconstructions of these proteins produce coherent clusters of purely eukaryotic homologues. Roughly one-tenth of the mitochondrial proteome can be traced with confidence to the α-proteobacteria. We infer that the genome of the ancestral α-proteobacterium that intitiated the mitochondrial lineages has lost most of its genes during the evolution of the organelle. In contrast, a majority of the genes encoding proteins of the modern mitochondrion are recruited from genes that evolved in the eukaryotic nucleus.

### Materials and methods

### Sequences

Yeast protein sequences putatively coding for mitochondrial proteins were extracted from public databases using the Gene Index numbers from a list of 423 proteins annotated as mitochondrial in the Yeast Protein Database (YPD, http://www.proteome.com; Hodges *et al*., 1999). The BLAST programme (Altschul *et al*., 1997) was used to search for homologous sequences in the mitochondrial genome of *Reclinomonas americana* (http://megasun.bch.umontreal.ca/ogmp/projects/other/mtcomp.html), WormPep17 (ftp://ftp.sanger.ac.uk/pub/databases/wormpep/), SwissProt and the non-redundant database at NCBI (nr). Homologous protein sequences were extracted and aligned using CLUSTAL W (Thompson *et al*., 1994). A fairly stringent BLAST cut-off value (E < 1e-10) was used in the inference of homology to avoid false positives. However, we find that the results change only marginally by lowering the cut-off value to E < 1e-5 (data not shown). Yeast paralogues were identified using the BLAST programme with the yeast genome as the database. Hits with an alignment score larger than 25% of the score for the query sequence against itself were defined as paralogues.

### Phylogenetic reconstructions

Phylogenetic trees were automatically reconstructed for all alignments with a significant number of homologues. The evolutionary relationships of the yeast mitochondrial proteins were estimated by the maximum-parsimony (MP), maximum-likelihood (ML) and neighbour-joining (NJ) methods (Saitou and Nei, 1987) using the programmes CLUSTAL W (Thompson *et al*., 1994), nj_plot, PAUP* (Swofford, 1999), phylo_win (Galtier *et al*., 1996) and TREE-PUZZLE (Strimmer and von Haeseler, 1996). Bootstrap values (MP, NJ) and puzzling steps (ML) for the trees were obtained from 100 (MP) and 1000 (ML, NJ) trees generated by random resampling of the data. The final selection of lineages to be included in the phylogenetic reconstructions were performed by manual inspection of the trees. The phylogenetic trees are available under 'other resources' in the *Comparative and Functional Genomics* HomePage (a section of *Yeast*) at **http://www.interscience.wiley.com**.

### Results

We have analysed a data set of 423 yeast genes that are annotated to code for mitochondrial proteins, 30 of which are encoded by the mitochondrial genome (Hodges *et al*., 1999. Homologous proteins were extracted from public databases and phylogenetic trees were reconstructed from all alignments with a significant number of homologous proteins, as schematically illustrated in Figure 1.

We observe that only half of the 423 yeast mitochondrial proteins (50.6%) have homologues in prokaryotes (E < 1e-10). Of these, 108 genes (25.5%) are represented in bacteria, archaea as well as eukaryotes (E < 1e-10); these may have been present in the last universal common ancestor. In addition, we have identified a set of 208 mitochondrial proteins (49.2%) with neither eubacterial nor archaeal homologues (E < 1e-10), 19 of which are encoded in the mitochondrial genome. Fifty-eight proteins in this group (30.5%) have homologues in *Caenorhabditis elegans* (E < 1e-10), suggesting that these genes originated prior to the divergence of yeast and nematodes. Another 74 genes (17.5%) are so far unique to yeast (E < 1e-10), 10 of which are mitochondrially encoded. These may represent proteins that arose recently in evolution or have diverged too far to be identified in other species.

Copyright © 2000 John Wiley & Sons, Ltd. 

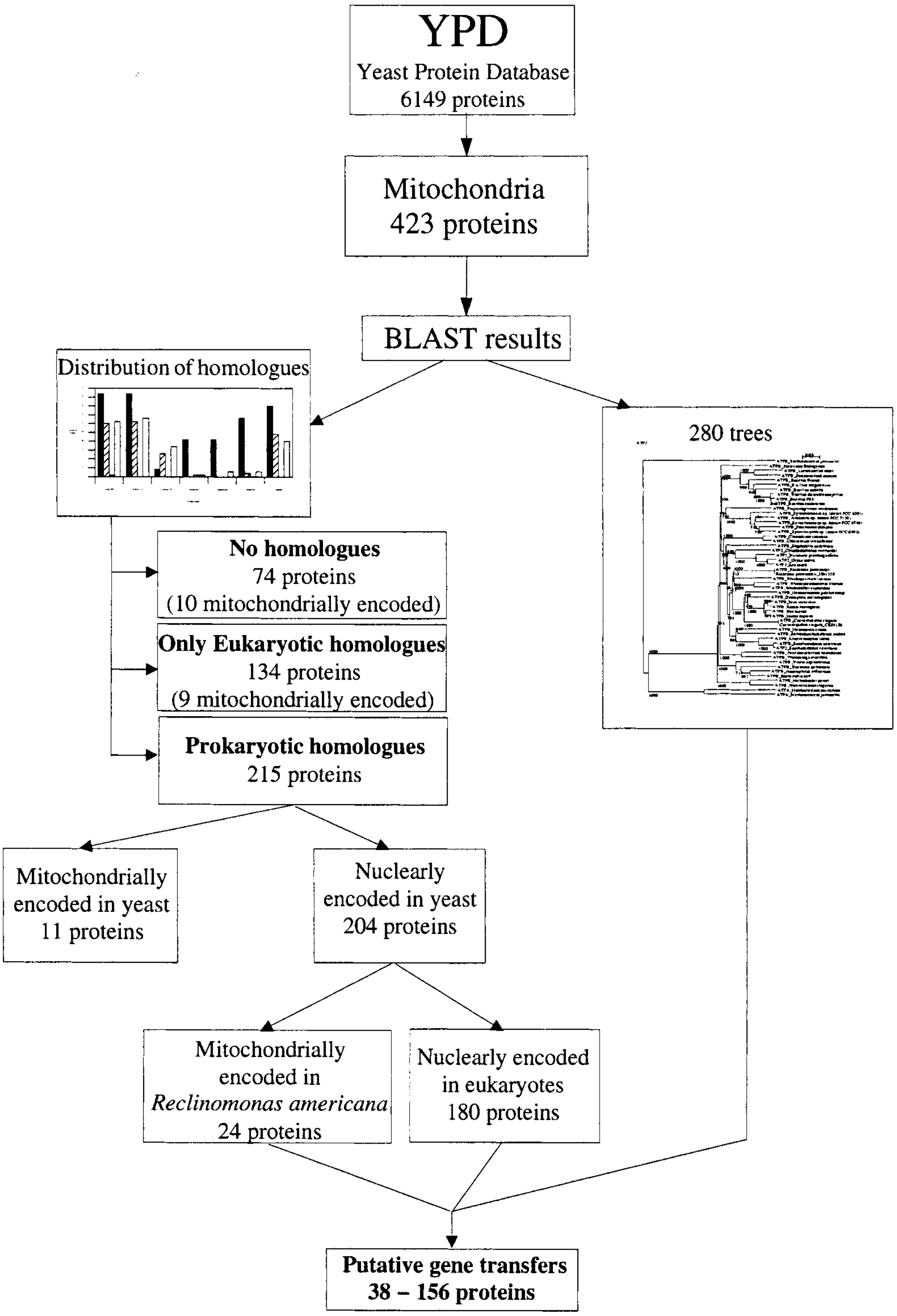

**Figure 1.** Schematic illustration of the strategy used for the analysis of the yeast mitochondrial proteome

Copyright © 2000 John Wiley & Sons, Ltd.

The relative fraction of yeast mitochondrial proteins with homologues in bacteria, archaea and eukaryotes varies markedly between the different functional categories, as indicated in Figure 2. For example, a majority of the proteins involved in bioenergetic (56.0%), translational (57.7%) and biosynthetic (88.2%) processes were found to have bacterial homologues (E < 1e-10). In contrast, bacterial homologues could be identified only rarely (E < 1e-10) for proteins involved in categories such as membrane (4.3%), regulation (12.5%) and transport (13.2%) (Figure 2). These distributions are consistent with the interpretation that mitochondrial transport and control functions were later additions to the core of bacterial genes that support respiration and gene expression.

### Mitochondrial protein complexes of bacterial origin

To study the origin and evolutionary history of the yeast mitochondrial proteins in greater detail we analysed our data set using phylogenetic methods. The sequence data, the alignments and the phylogenetic reconstructions based on neighbour-joining, maximum parsimony and maximum likelihood methods are available under 'other resources' in the *Comparative and Functional Genomics* HomePage (a section of *Yeast*) at **http://www.interscience.wiley.com**.

The relationship between mitochondrial genomes and α-proteobacteria is already well supported by phylogenetic analyses of bioenergetic proteins such as cytochrome *b* and cytochrome *c* oxidase subunits (Andersson *et al.*, 1998; Sicheritz-Pontén *et al.*, 1998; Gray *et al.*, 1999). There is little doubt that a majority of the mitochondrion-encoded genes are derived from α-proteobacterial ancestors. Nevertheless, out of more than 100 mitochondrial proteins involved in bioenergetic processes, only seven are encoded by the yeast mitochondrial genome; the remainder are encoded by the nuclear genome.

In order to search for genes that are likely to have been transferred from the endosymbiont/mitochondrion into the nuclear genome, we ana-

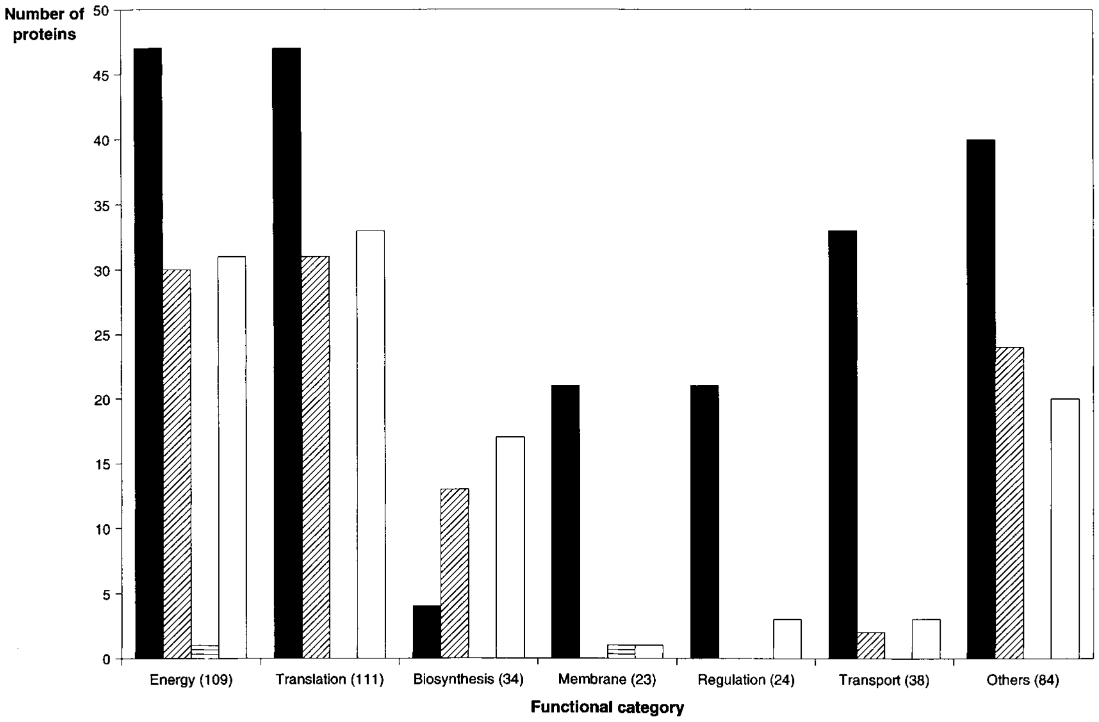

**Figure 2.** Histogram representation of the similarity of yeast mitochondrial proteins to their homologues in bacteria, archaea and eukaryotes (E < 1e-10). The bars represent yeast proteins with homologues to eukaryotes only (black bars), to eukaryotes and bacteria only (diagonal lines), to eukaryotes and archaea only (horisontal lines) and to eukaryotes, bacteria and archaea (white bars)

Copyright © 2000 John Wiley & Sons, Ltd.

lysed a set of 24 homologous proteins that are encoded by the nuclear genome of yeast as well as by the mitochondrial genome of *Reclinomonas americana*. This is hypothesized to be the most primitive mitochondrial genome with the largest protein-coding capacity known for this organelle (Lang *et al.*, 1997). Half of the genome encodes proteins involved in information processes and the other half encodes proteins mediating bioenergetic processes. Phylogenetic reconstructions based on a combined dataset of 10 ribosomal proteins show that the nucleus-encoded yeast genes cluster with their mitochondrion-encoded homologues in *R. americana* (Figure 3A). Ribosomal protein genes are highly clustered in bacterial genomes as well as in the mitochondrial genome of *R. americana*. Accordingly, it seems likely that the entire ensemble of ribosomal protein genes were retained in the genomic precursor of mitochondria during the initial endosymbiosis. At later stages, different subsets of the ribosomal protein genes seem to have been transferred into the nuclear genomes of different eukaryotic lineages. The transferred genes have been extensively shuffled around and only two ribosomal protein genes are located near to each other in the modern yeast nuclear genome. These code for ribosomal proteins S12 and S19, which are located in the *str* and S10 ribosomal protein gene operons in bacterial genomes.

Likewise, phylogenetic reconstructions for components of the succinate dehydrogenase complex (Figure 3B) illustrate the close phylogenetic relationship between the mitochondrial genes in *R. americana* and their nuclear-encoded homologues in other eukaryotes (Burger *et al.*, 1996). In addition, a tree reconstructed from the concatenated align-

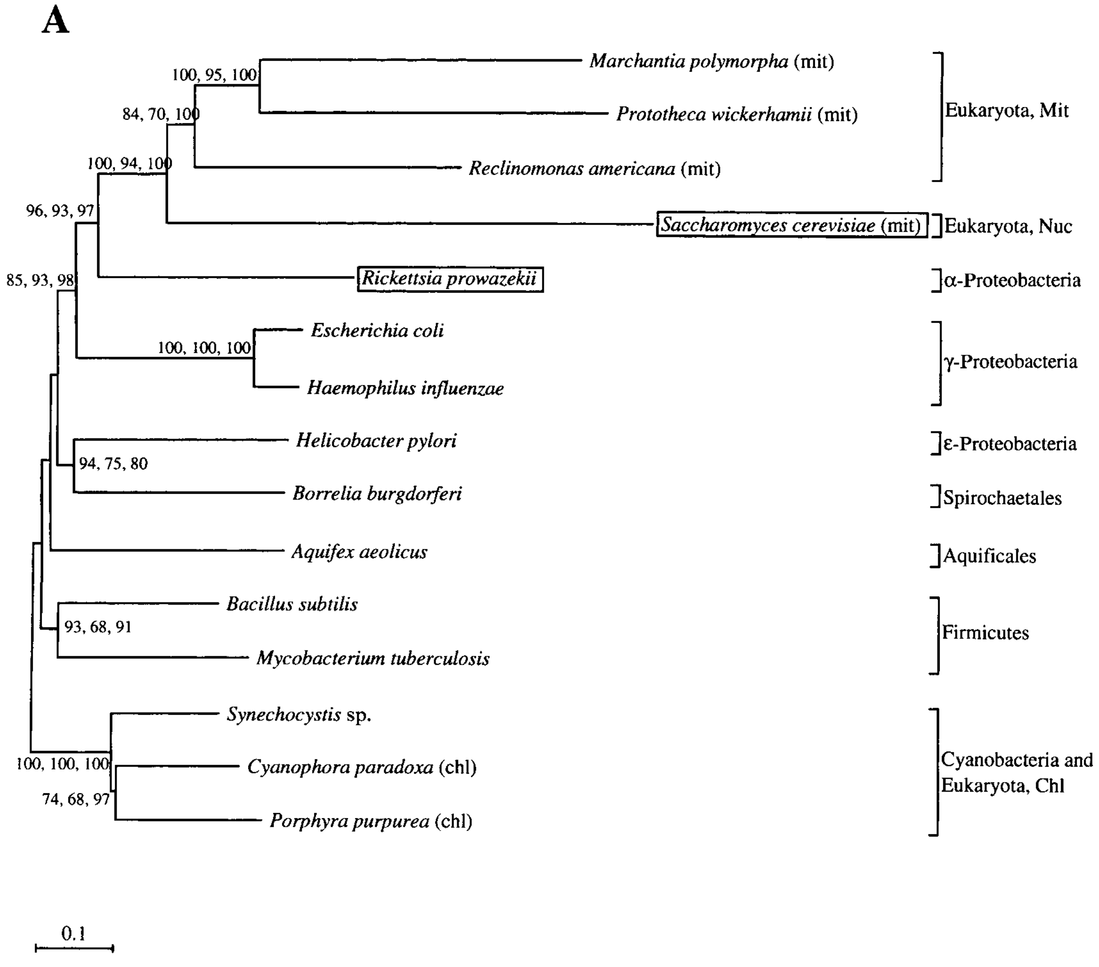

**Figure 3a.**

Copyright © 2000 John Wiley & Sons, Ltd. 

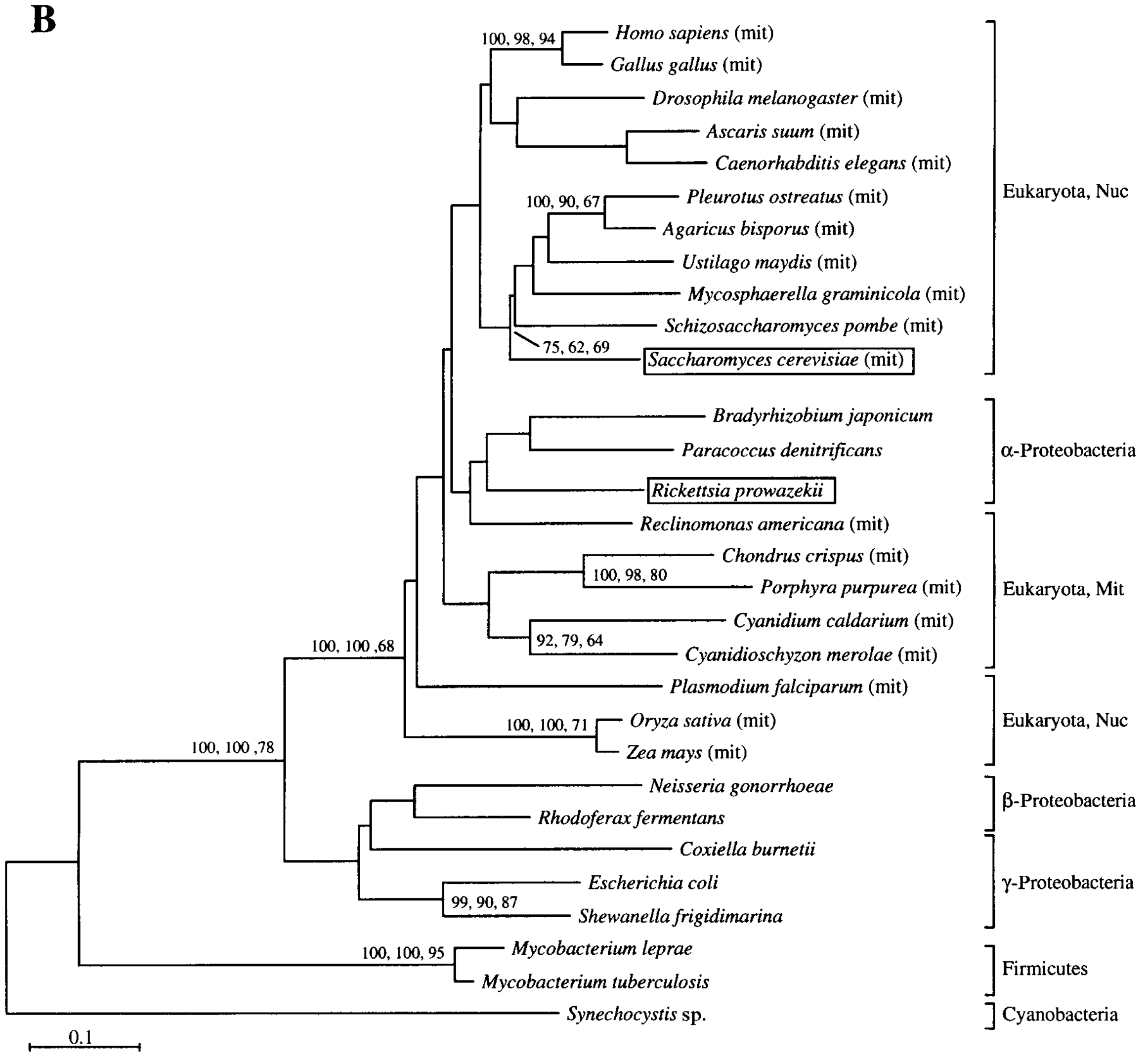

**Figure 3b.**

ments of the α and γ subunits of the ATP synthase complex generates a distinct mitochondrial clade (Figure 3C). It is worth emphasizing that these homologues are located in the nuclear genome of yeast, but are found in the mitochondrial genome of *R. americana*. We conclude that most of the 24 yeast mitochondrial proteins with homologues in *R. americana* were present in the ancestral α-proteobacterium and have been transferred into the nuclear genome of yeast via the endosymbiont/mitochondrion genome intermediate.

At least 14 additional genes that are nuclear in all eukaryotes are also strong candidates for ancient gene transfers from α-proteobacteria to nuclear genomes. For example, phylogenetic reconstructions obtained with the mitochondrial pyruvate dehydrogenase (PDH) subunits that are encoded by nuclear genes suggest that these are descendents of an α-proteobacterial ancestor from which *Rickettsia* is also derived (Figure 4). It should be noted that there are two paralogous genes coding for the dihydrolipoamide dehydrogenase E3 component in *R. prowazekii*, only one of which was transferred into the nuclear genome of the eukaryotes (Figure 4C). We infer that a minimum of 38 genes (ca. 10%) have been successfully transferred into the nuclear genome of yeast. Most of these encode proteins in the bioenergetic and translation complexes.

Copyright © 2000 John Wiley & Sons, Ltd.

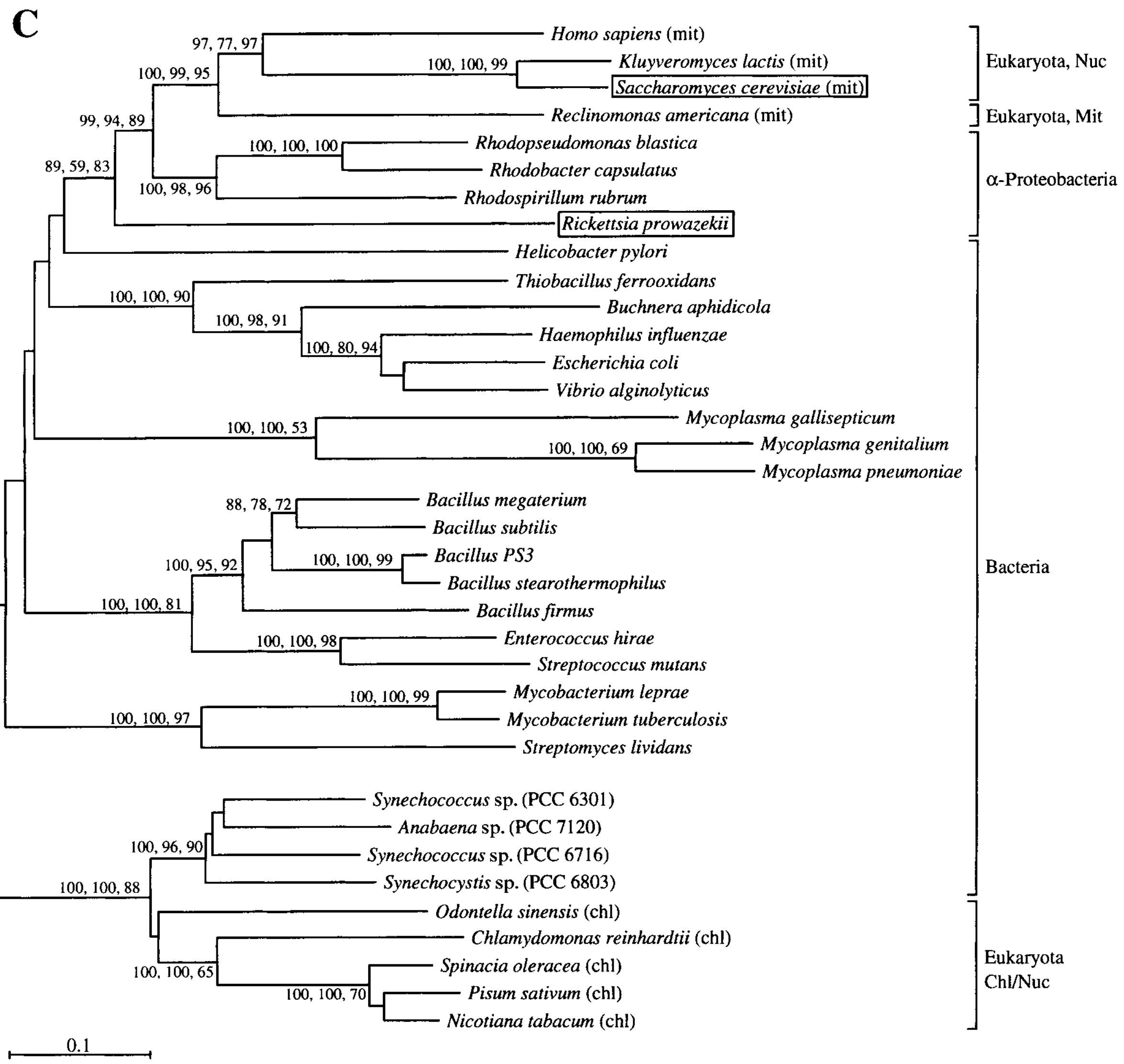

**Figure 3.** Examples of mitochondrial proteins of α-proteobacterial orgin that are encoded by the nuclear genome of *Saccharomyces cerevisiae* and the mitochondrial genome of *Reclinomonas americana*. The phylogenetic trees were constructed from: (A) the combined protein sequences of the ribosomal proteins S2, S7, S10, S12, S13, S14, S19, L5, L6 and L16; (B) the succinate dehydrogenase iron sulphur protein; (C) the combined protein sequences of the α- and γ-subunits of the ATP synthase complex. Names outside brackets refer to the genome encoding the protein (Mit = mitochondrial genome; Nuc = nuclear genome; Chl = chloroplast genome). Names in parentheses refer to the location of the protein (mit = mitochondria; chl = chloroplast). Branch lengths are proportional to those reconstructed with the NJ method. Values at nodes indicate the percentage of 1000 NJ bootstraps, 100 MP bootstraps and 1000 ML puzzling steps, in this order

### Mitochondrial protein complexes of mixed origin

A more detailed inspection of individual mitochondrial functional complexes shows that bacteria-derived genes typically encode core components of these complexes. However, many species-specific subunits have subsequently been added to these putative enzyme cores. These species-specific components have most likely originated by the recruitment of novel genes from within the nuclear genomes. For example, the core components of the cytochrome *bc*1 complex (cytochrome *b*, cytochrome *c*1 and the Rieske iron–sulphur protein) cluster

Copyright © 2000 John Wiley & Sons, Ltd.

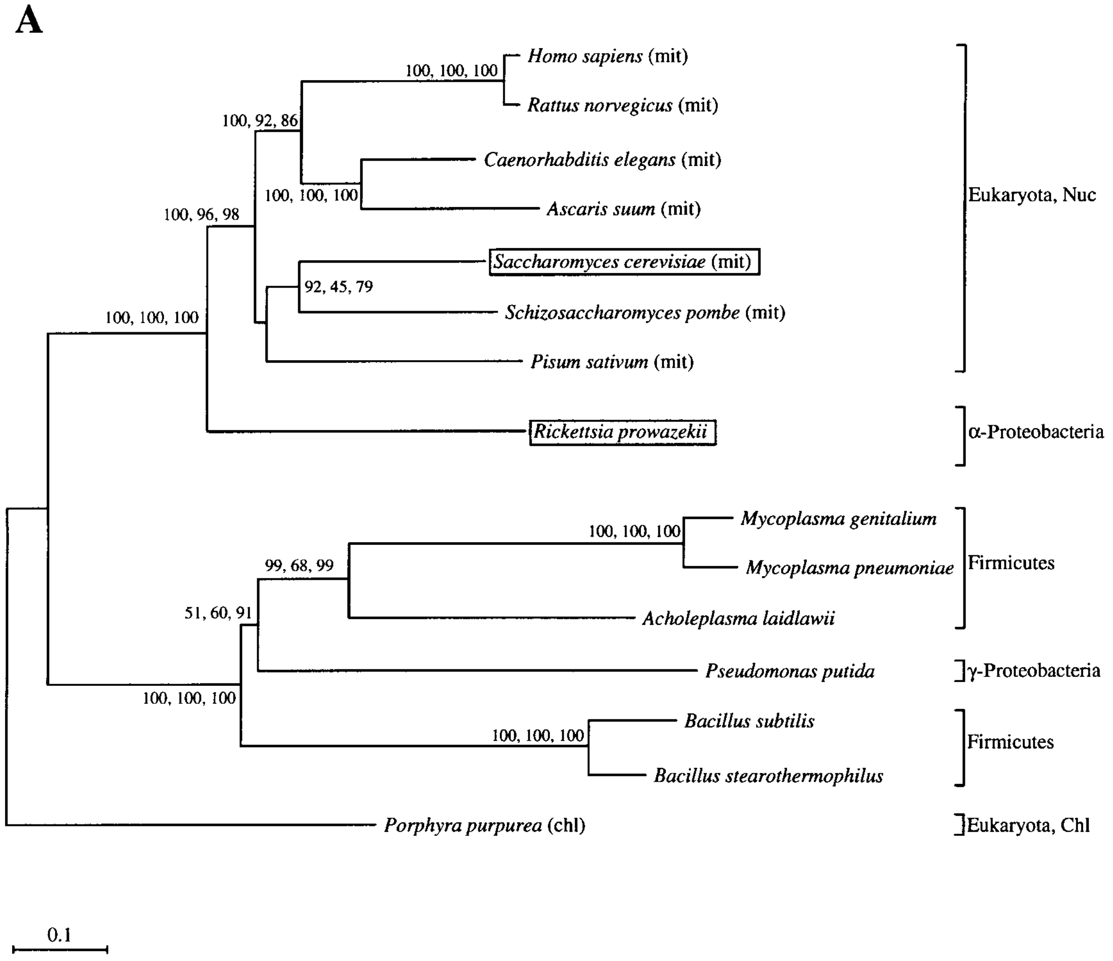

**Figure 4a.**

closely with their α-proteobacterial relatives (Sicheritz-Pontén *et al.*, 1998). In contrast, seven other subunits of the yeast mitochondrial cytochrome *bc*1 complex have no bacterial homologues. The core components of the cytochrome *c* oxidase complex (cytochrome *c* oxidase subunits I, II and III) are derived from α-proteobacteria (Sicheritz-Pontén *et al.*, 1998; Gray *et al.*, 1999), while most of the proteins in this complex have no bacterial homologues. Several additional proteins are involved in the assembly of this complex, only two of which have bacterial homologues (*cox*11 and *cox*15).

Finally, we note that even the mitochondrial ribosome contains proteins that are of α-proteobacterial origin (Figure 3A) as well as others that are similar only to eukaryotic homologues encoded in nuclear genomes. Approximately 60% of the mitochondrial ribosomal proteins have recognizable bacterial homologues. This provides a minimal estimate of the fraction of mitochondrial ribosomal proteins of putative eubacterial origin. Some ribosomal proteins are very short, which makes inferences of shared ancestry based on sequence similarities very difficult. In any case, the yeast mitochondrial ribosome consist of about 10 more proteins than the bacterial ribosome. Thus, the fraction of ribosomal proteins of eukaryotic origin is likely to be at least 15% and at most 40%.

### Mitochondrial protein complexes of nuclear origin

Proteins involved in regulation and transport processes tend to be more exclusively of eukaryotic origin. For example, all yeast mitochondrial compo-

Copyright © 2000 John Wiley & Sons, Ltd. 

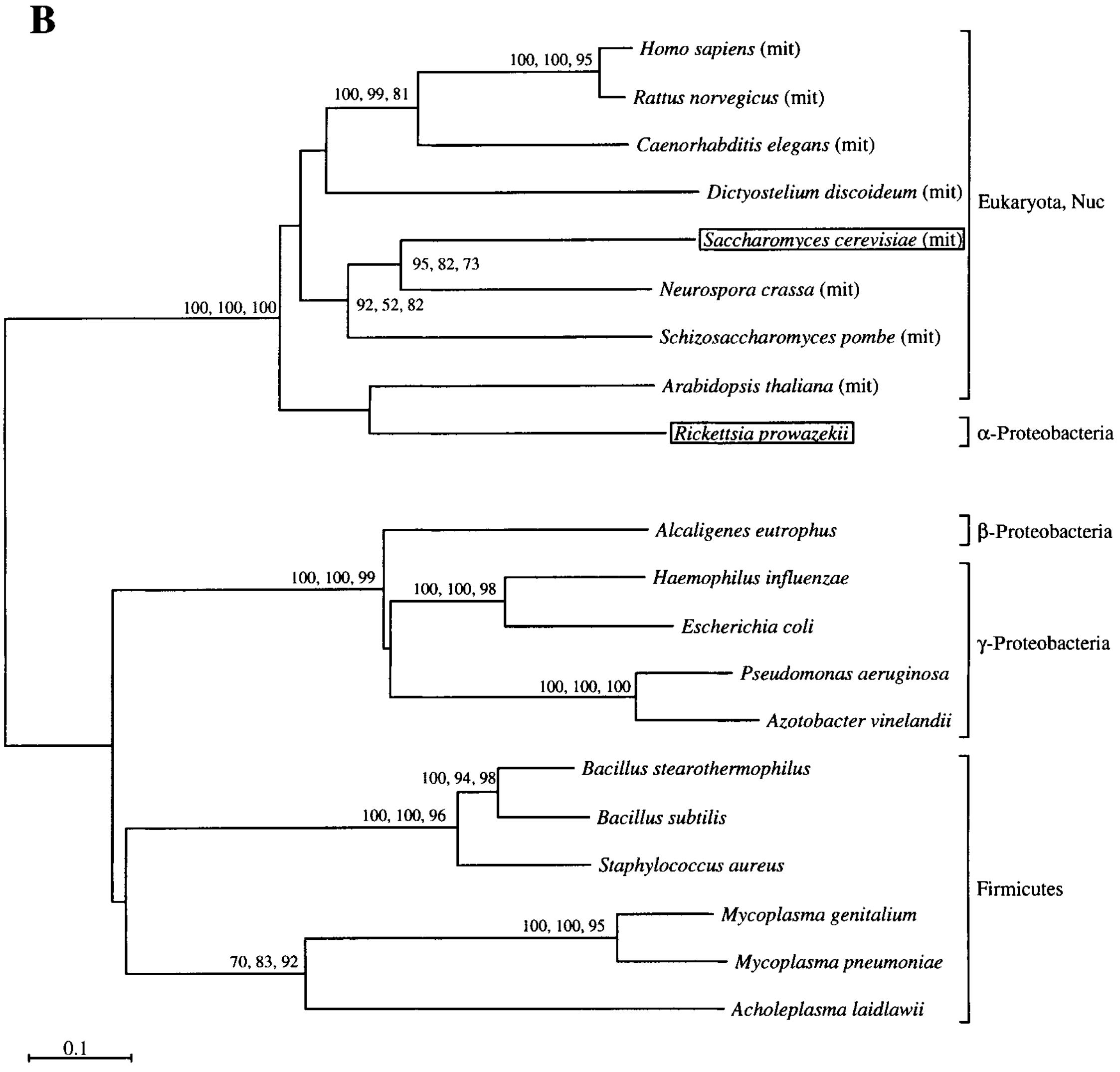

**Figure 4b.**

nents in protein complexes associated with the regulation of gene expression, mRNA stability and splicing seem to be purely of eukaryotic origin. The eukaryotic descent of these proteins is inferred from an almost complete absence of bacterial homologues for these functions. Likewise, many membrane and transport complexes appear to be derived exclusively from the eukaryotic clusters. These include the TIM and TOM family of membrane proteins that facilitate protein import across the mitochondrial membrane, as well as the ATP/ADP translocases that shuffle ATP and ADP across the membrane (Figure 5). Both of these represent highly specialized transport functions that would not be found in free-living bacteria (Andersson, 1998; Andersson *et al.*, 1998; Andersson and Kurland, 1999). In the present study we could not discover any significant sequence similarities to bacterial proteins for these membrane proteins. We infer that such proteins evolved after the integration of the endosymbiont/mitochondrion into the eukaryotic cell.

### Duplication and divergence

To examine the relative fraction of mitochondrial proteins associated with gene and genome duplications, we searched the entire yeast genome for sequence similarities using mitochondrial proteins as queries. In total, we identified 61 paralogous gene groups (Figure 6). Approximately half of these

Copyright © 2000 John Wiley & Sons, Ltd. 

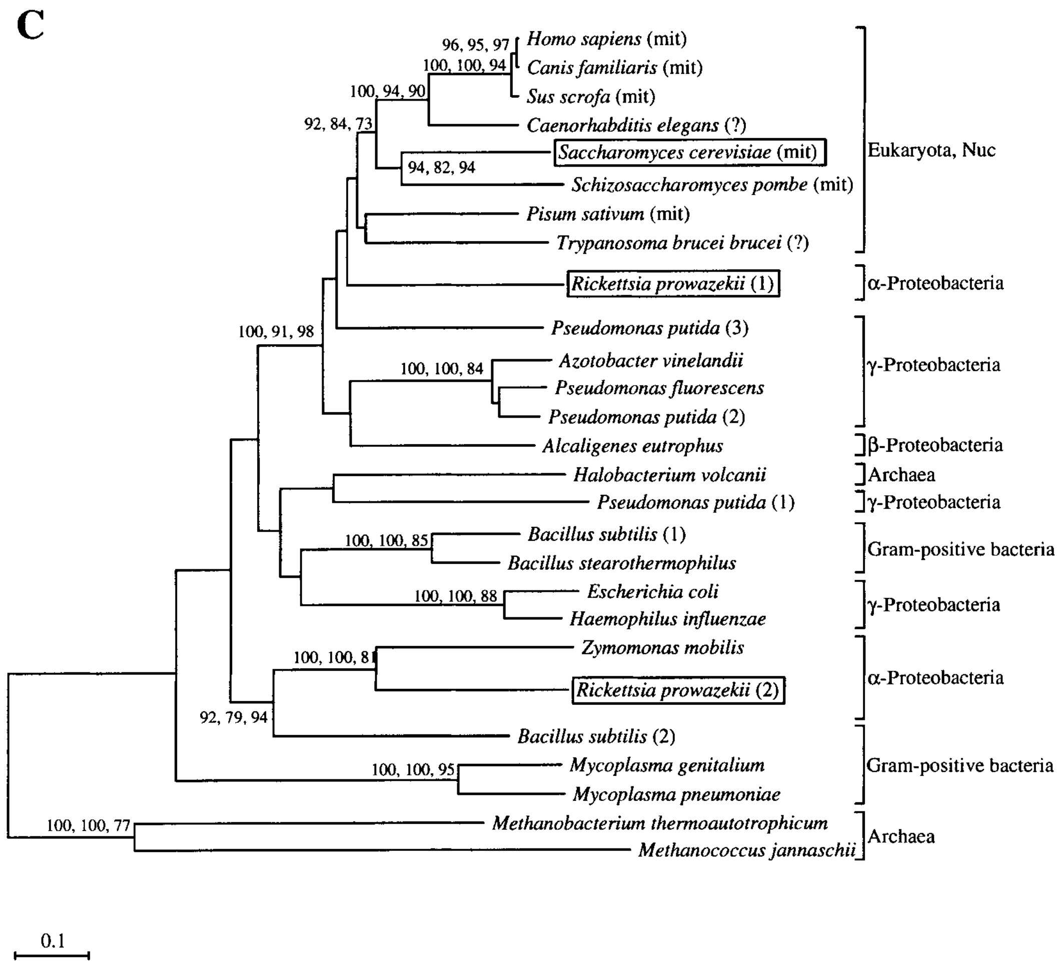

**Figure 4.** Examples of mitochondrial proteins of $\alpha$-proteobacterial origin that are encoded by the nuclear genome of *Saccharomyces cerevisiae* and other eukaryotes. The phylogenetic trees were constructed from: (A) the combined protein sequences of the $\alpha$- and $\beta$-subunits of the pyruvate dehydrogenase E1 component; (B) the dihydrolipoamide acetyltransferase E2 component; (C) the dihydrolipoamide dehydrogenase E3 component. Names outside brackets refer to the genome encoding the protein (Mit = mitochondrial genome; Nuc = nuclear genome; Chl = chloroplast genome). Names in parentheses refer to the location of the protein (mit = mitochondria; chl = chloroplast). Branch lengths are proportional to those reconstructed with the NJ method. Values at nodes indicate the percentage of 1000 NJ bootstraps, 100 MP bootstraps and 1000 ML puzzling steps, in this order duplications have been suggested to have resulted from a putative whole genome duplication event (Wolfe and Shields, 1997; Seoighe and Wolfe, 1999), whereas the other half seems to be the result of single gene duplication events. This fraction is similar for gene pairs that encode two mitochondrial proteins vs. those that encode one protein targeted to the mitochondrion and the other to the cytoplasm. Most of the genes in these groups are clustered with their duplicated gene copy in phylogenetic reconstructions.

### Re-targeting and displacement

To be functional in the mitochondrion, a gene transferred from the organelle's genome to the nucleus often requires an appropriate targeting signal appended to the transferred gene. However, if a similar cytoplasmic gene function is already available in the genome, switching the cytoplasmic for a mitochondrial targeting signal is required in order to recruit the protein to the organelle. Gene duplications would facilitate the recruitment of

Copyright © 2000 John Wiley & Sons, Ltd. 

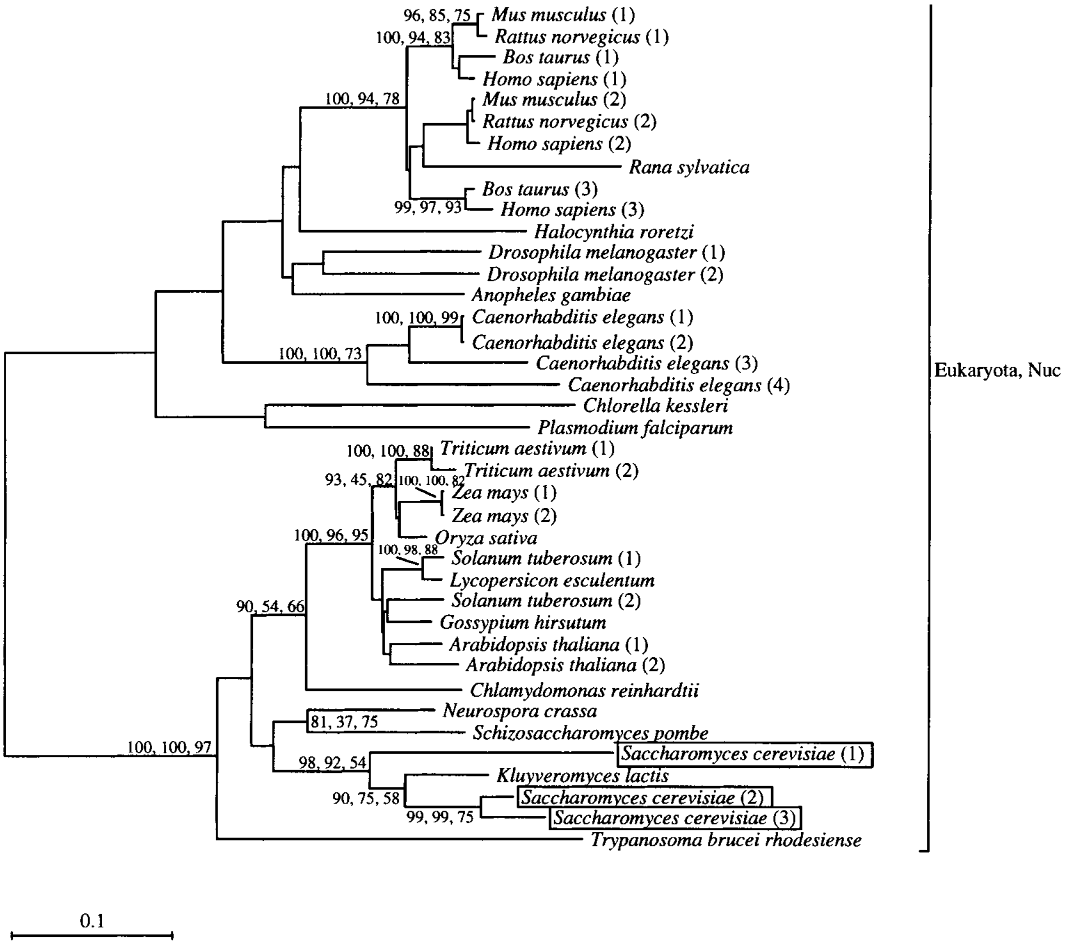

**Figure 5.** Examples of mitochondrial proteins of eukaryotic origin. The phylogenetic trees were constructed from the mitochondrial ADP/ATP translocases in eukaryotes. Branch lengths are proportional to those reconstructed with the NJ method. Values at nodes indicate the percentage of 1000 NJ bootstraps, 100 MP bootstraps and 1000 ML puzzling steps, in this order cytoplasmic proteins to the organelle, or vice versa, by permitting separate targeting to different compartments for the protein products.

For example, many proteins involved in the TCA cycle are associated with complex evolutionary histories that involve both duplications and retargeting. One enzyme, malate dehydrogenase, appears to have been associated with at least three gene duplication events in yeast. Each of the three modern copies is targeted to a different compartment: the mitochondrion, the cytoplasm and the peroxisome (Figure 7A). Aconitase hydratase is another component of the TCA cycle that is required in both the mitochondrion and the cytoplasm. However, in this case it is the nuclear genes encoding the cytoplasmic enzymes that are most similar to the bacterial proteins (Figure 7B).

The aminoacyl-tRNA synthetases represent yet another family of enzymes that have distinguishable origins in both the bacterial and in the eukaryotic genomes. This complex protein family can evolve in eukaryotes in either of two ways. One is by maintaining both bacterial and eukaryotic lineages of enzymes in separate cell compartments. The other is by replacement of an enzyme from one of the lineages by its homologue in the other. We find that as many as 12 aminoacyl-tRNA synthetases are encoded by two genes with different ancestries, one of which code for the mitochondrial and the other for the cytoplasmic protein. For example, the cytoplasmic glutamyl-tRNA synthetase seems to be derived from within the nuclear genome, whereas the mitochondrial glutamyl-tRNA synthetase seems to be bacterially derived (Figure 8A). The other

Copyright © 2000 John Wiley & Sons, Ltd. 

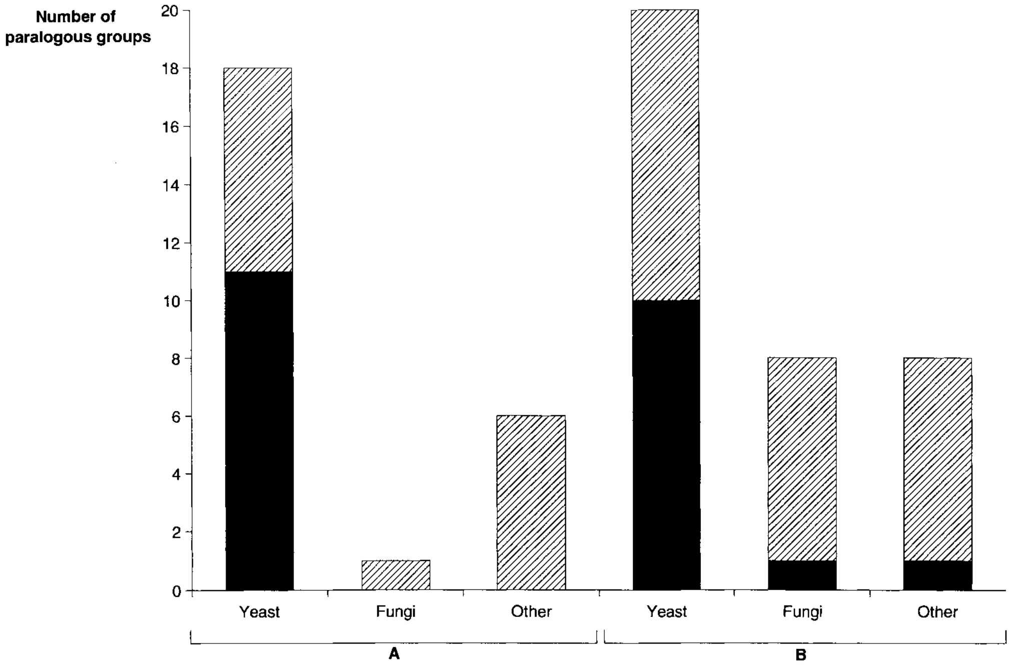

**Figure 6.** Histogram representation of the fraction of paralogous gene clusters coding for (A) two or more proteins expressed in the mitochondria, (B) one protein expressed in the mitochondrion and the other in the cytoplasm. The bars represent gene duplication events thought to have originated by the complete genome duplication event (black bars) or by individual gene duplication events (hatched bars). The relative fraction of paralogous gene clusters with homologues in yeast, fungi and other eukaryotes are shown mitochondrial synthetases in this class are also in general more similar to the bacterial synthetases than to their cytoplasmic homologues, but they cluster only rarely with the α-proteobacteria. Moreover, *Rickettsia*, which is our principal reference for the α-proteobacteria, often occupies atypical positions in phylogenetic trees based on the aminoacyl-tRNA synthetases. This makes it difficult to accurately assess the relationship between the mitochondrial and the α-proteobacterial synthetases.

Another class of 3 aminoacyl-tRNA synthetases have more complex origins. These are found as similar, multiple copies of genes in either the mitochondrial or the cytoplasmic lineage. Such a pattern suggests that they arose as relatively recent gene duplications in one lineage and that homologues from the other lineage were lost. For example, the mitochondrial and the cytoplasmic arginyl-tRNA synthetases cluster closely together in phylogenetic reconstructions (Figure 8B). Still another class of 4 amino acyl-tRNA synthetases are encoded by a single gene, which presumably serves both mitochondrial and cytoplasmic functions. The histidyl-tRNA synthetase is here used as a representative of this class of enzymes (Figure 8C).

### Discussion

The phylogenetic reconstructions summarized here suggest that the proteome of the yeast genome is composed of descendants of diverse genomic ancestors. These include a minority group of the expected descendants of the α-proteobacteria that presumably were introduced by the endosymbiotic ancestor of the mitochondria. However, they also include an unexpected major group of eukaryotic descendants with no apparent bacterial antecedents. In addition, there is a small cohort of proteins of unspecified bacterial origin, some or all of which may have been introduced from bacteria other than the ancestral endosymbiont. Difficulties in identifying the specific bacterial subdivision from which this group arose may be due to a variety of factors. These include

Copyright © 2000 John Wiley & Sons, Ltd. 

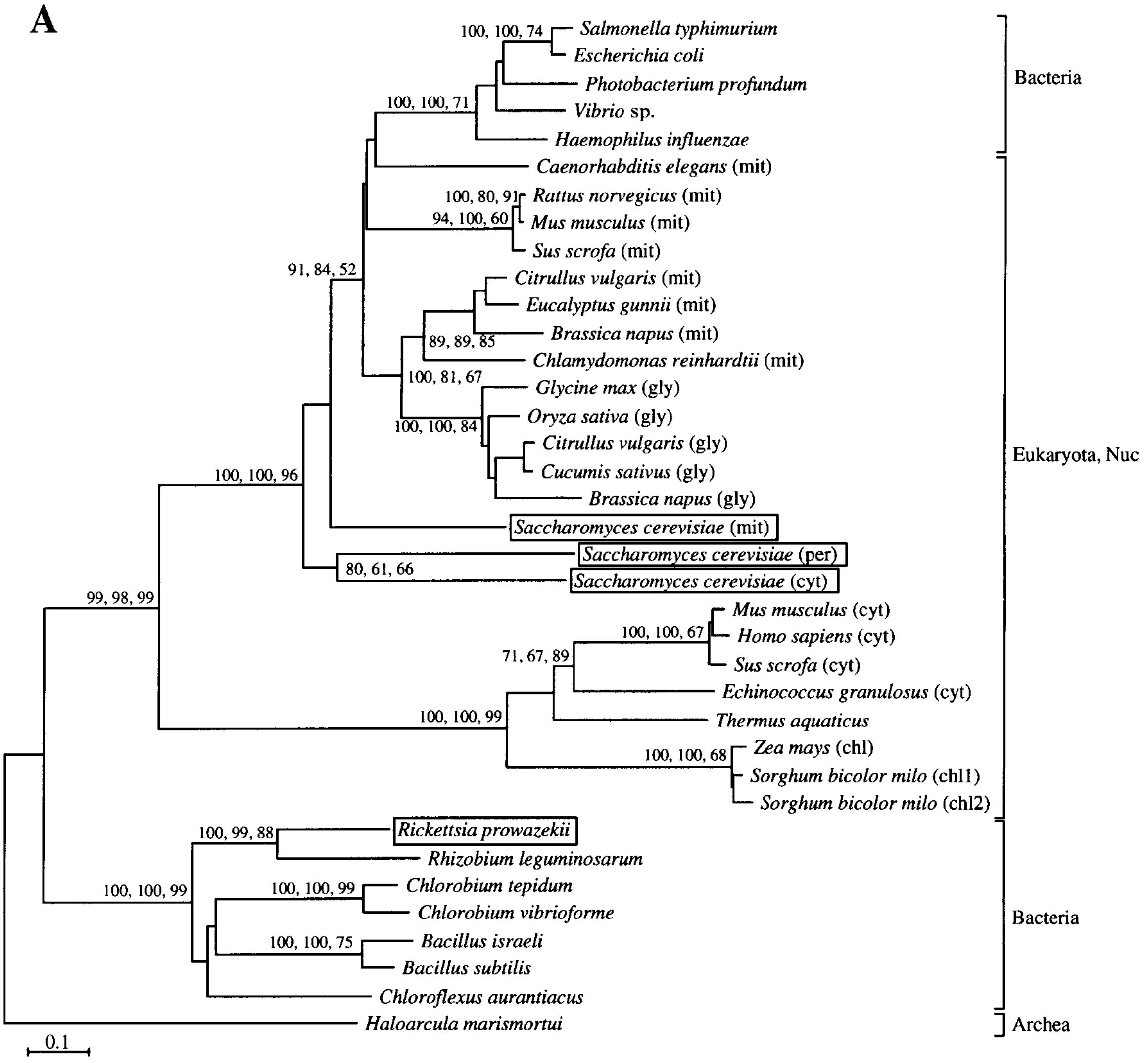

**Figure 7a.**

ambiguities introduced by rapid rates of sequence evolution, short protein lengths, the presence of paralogous genes, and/or a diversity of bacterial origins.

What does the phylogenetic diversity of the mitochondrial proteome tell us about the evolution of mitochondria? The import from the cytosol of most mitochondrial proteins is dependent on a specific, complex transport system, most elements of which are not found in free living $\alpha$-proteobacteria (Schatz, 1996; Neupert, 1997). This means that characteristic functions of mitochondria, such as protein import, must have evolved after the initial symbiotic relationship had been established. The acquisition of novel functions may have involved recruiting bacterial proteins for new mitochondrial functions. Examples of this mode are the chaperonin proteins that are involved in the import of proteins into mitochondria synthesized in the cytosol (Viale and Arakaki, 1994; Schatz, 1996; Neupert, 1997). An alternative evolutionary route is to recruit proteins from the eukaryotic nuclear genome for service in the organelle (Andersson and Kurland, 1999). We infer that the major group of mitochondrial proteins identified here as eukaryotic in origin represent just such nuclear gene products that have been recruited to the organelle. This group includes proteins other than the chaper-

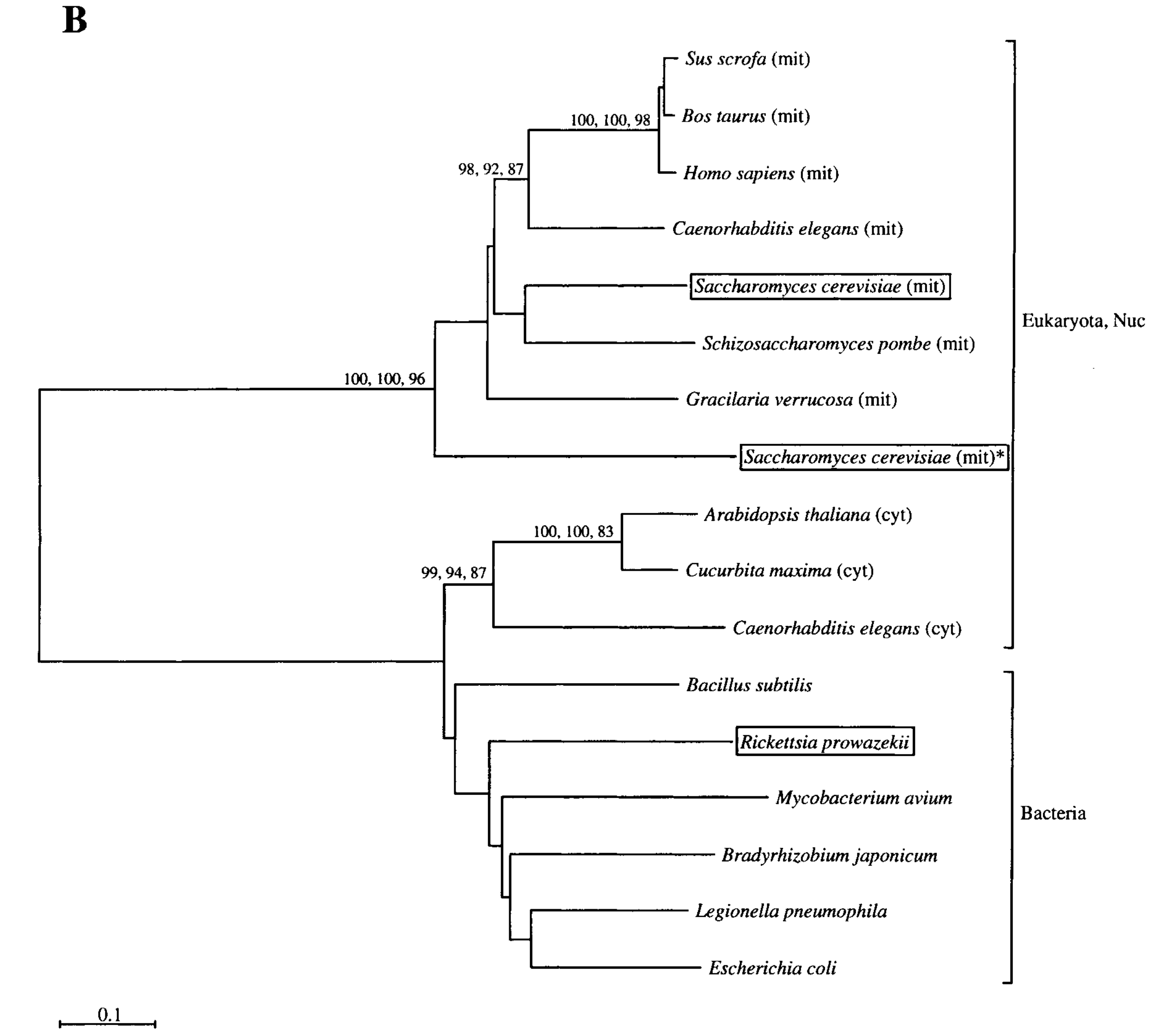

**Figure 7.** Examples of mitochondrial proteins encoded by duplicated genes. The phylogenetic trees were constructed from (A) malate dehydrogenase, (B) aconitase hydratase. *Protein with similarity to aconitase, has a potential mitochondrial transit peptide. Names outside brackets refer to the genome encoding the protein (Mit = mitochondrial genome; Nuc = nuclear genome; Chl = chloroplast genome). Names in parentheses refer to the location of the protein (mit = mitochondria; chl = chloroplast; cyt = cytoplasm; per = peroxisome; gly = glyoxysome). Branch lengths are proportional to those reconstructed with the NJ method. Values at nodes indicate the percentage of 1000 NJ bootstraps, 100 MP bootstraps and 1000 ML puzzling steps, in this order onins that participate in the protein transport system (Schatz, 1996; Neupert, 1997).

It seems that the mitochondrial proteome has evolved in two distinctive modes. One of these is a pronounced reductive mode in which nearly all the ancestral genes of the original symbiont have been discarded and only a small fraction has been retained, primarily in nuclear genomes (Andersson and Kurland, 1998). The present data suggest that this remnant corresponds to at least 38 proteins in yeast, as inferred from strong phylogenetic signals to the $\alpha$-proteobacteria. Evidence of a compensating expansive mode is found in the nearly 200 proteins that have been identified here as novel recruits from the eukaryotic genomes. We used a fairly stringent BLAST cut-off value (E < 1e-10) in the inference of homology, so the number of bacterial and eukaryotic homologues may be slightly underestimated.

Copyright © 2000 John Wiley & Sons, Ltd.

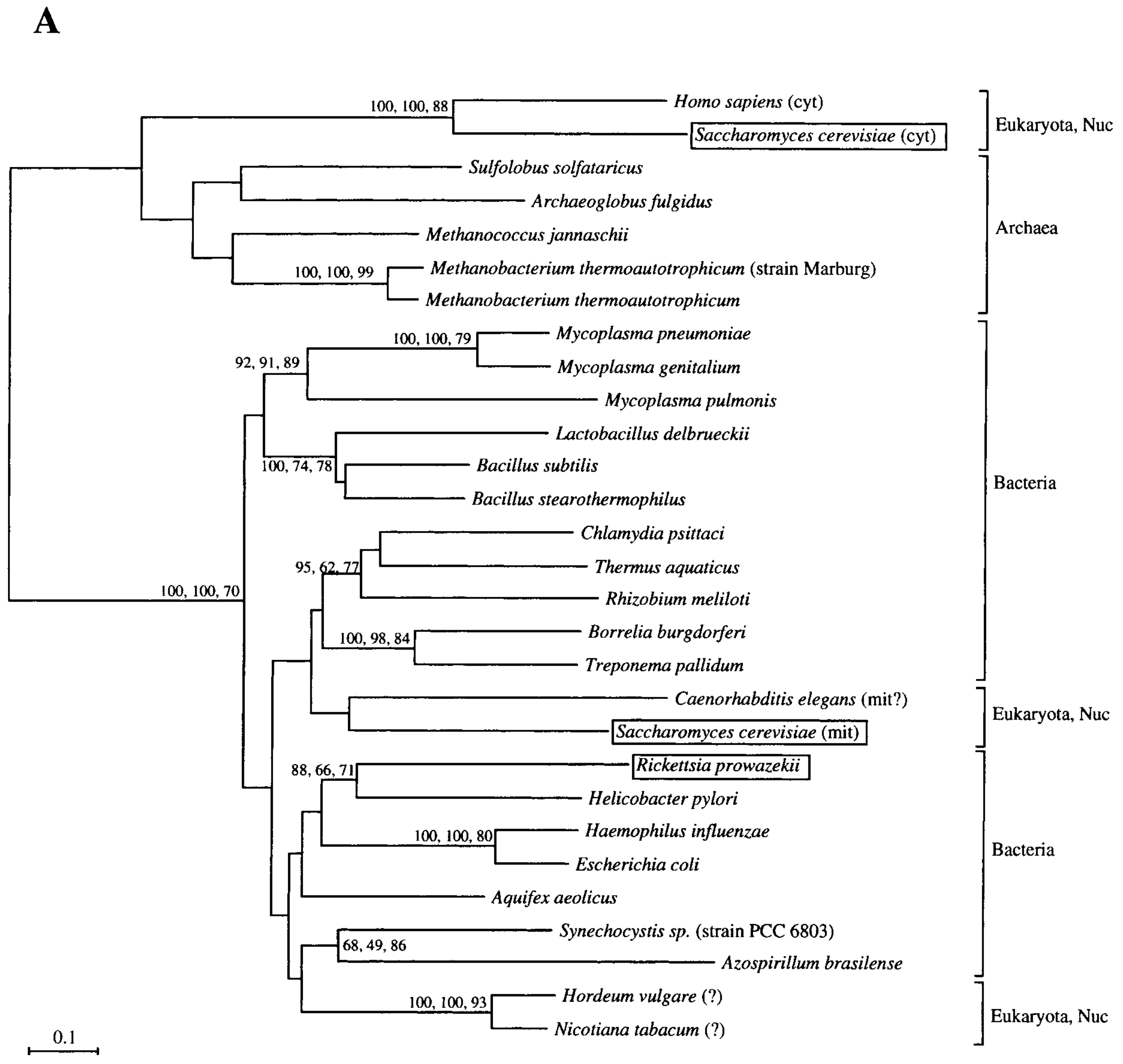

**Figure 8a.**

However, the results change only marginally by lowering the cut-off value to E<1e-5 (data not shown), so the fraction of unrecognized homologous proteins is likely to be very low.

The divergent ancestries of these classes of proteins is also reflected in their functional contributions (Andersson *et al.*, 1998). Thus, the core components of the respiratory system, as well as of the translational machinery of the mitochondria, are indeed derived from the ancestral symbiont. In contrast, a major fraction of other characteristic functions, such as transport and regulation, seems to have arisen in the nuclear genomes subsequent to the ancestral endosymbiotic event. This is consistent with the naive expectation that proteins mediating import and export across the mitochondrial membrane originated subsequent to the integration of mitochondria into the eukaryotic cell.

A weakness of the endosymbiotic hypothesis for the origin of mitochondria is that it does not explain what constituted the initial symbiosis between the ancestral $\alpha$-proteobacterium and its host (Andersson *et al.*, 1998; Martin and Müller, 1998; Gray *et al.*, 1999). This issue introduces another dimension to views of the evolution of the mitochondrial proteome. Some recent accounts of

Copyright © 2000 John Wiley & Sons, Ltd. 

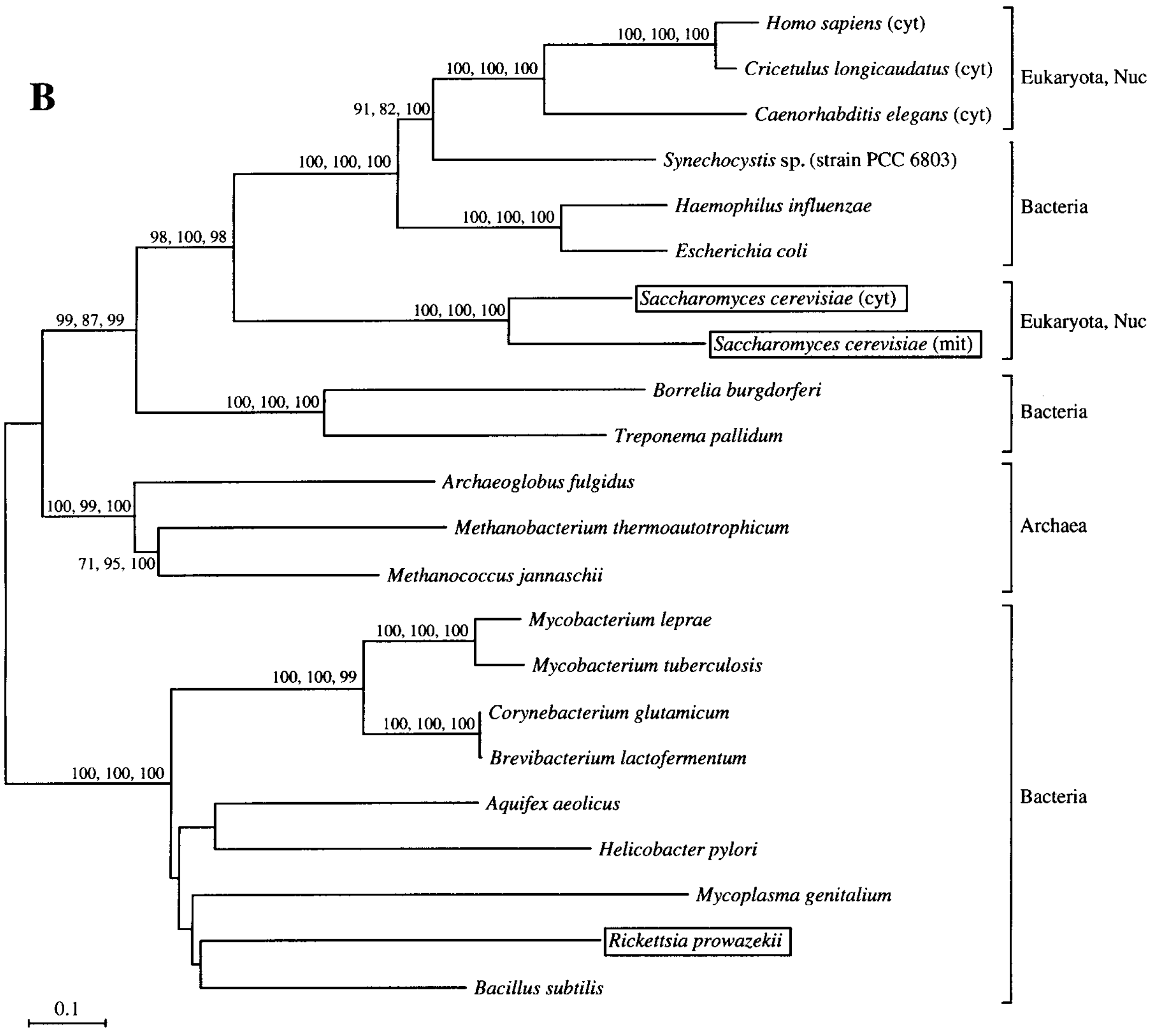

**Figure 8b.**

the origin of mitochondria have emphasized the anaerobic metabolic capacities of the putative α-proteobacterial ancestor (Martin and Müller, 1998; Lopez-Garcia and Moreira, 1999). Others have focused on the aerobic respiration of the putative α-proteobacterial ancestor (Fenchel and Finlay, 1995; Andersson and Kurland, 1999).

The fact that pyruvate is the essential substrate for hydrogen production by the anaerobic hydrogenosomes as well as for the Krebs cycle in aerobic mitochondria has been adduced as evidence for the close phylogenetic affinity of these two organelles (Martin and Müller, 1998). Nevertheless, the enzyme pyruvate ferredoxin oxidoreductase (PFO) is used by hydrogenosomes, while aerobic mitochondria utilize the unrelated enzyme, PDH.

Phylogenetic reconstructions based on mitochondrial subunits of the PDH complex reveal a close affiliation with the homologous proteins in the α-proteobacteria (Figure 4). Sequence data for both bacterial and eukaryotic PFOs were used to test the most straightforward expectation of the hydrogen hypothesis, namely, that the PFO of hydrogenosomes should also cluster with those of the α-proteobacteria. The available phylogenetic data do not support this prediction (Horner *et al.*, 1999). An alternative interpretation that the hydrogenosomes have been repeatedly and independently derived from mitochondria during transitions from aerobic to anaerobic environments is consistent with these observations (Embley *et al.*, 1997).

In any case, there is agreement that the free-living

Copyright © 2000 John Wiley & Sons, Ltd.

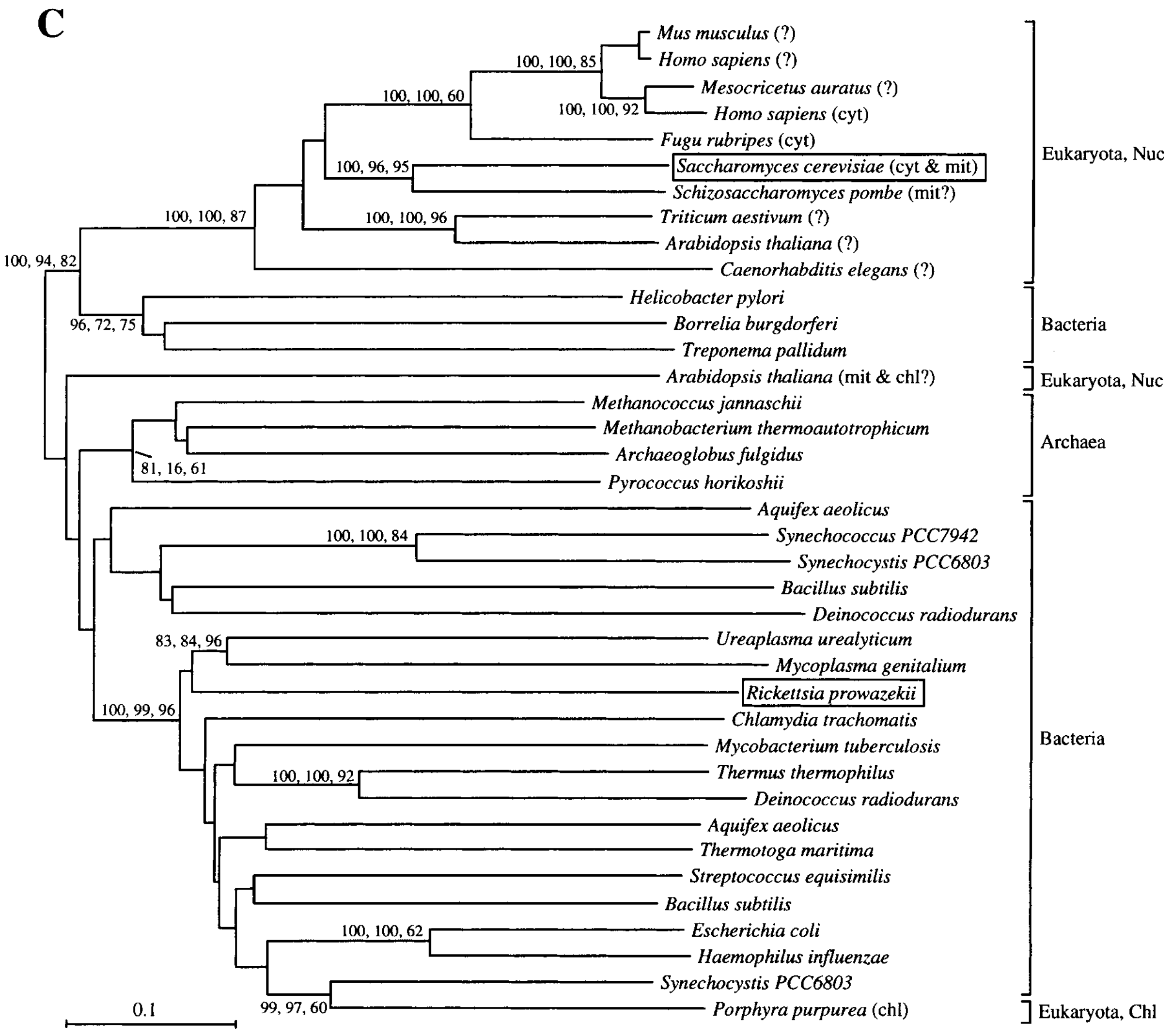

**Figure 8.** Examples of three different modes of evolution for the mitochondrial aminoacyl-tRNA synthetases. The phylogenetic trees were constructed from: (A) glutamyl-tRNA synthetase – the mitochondrial and the cytoplasmic homologues are encoded by genes of different origins; (B) arginyl-tRNA synthetase – the mitochondrial and the cytoplasmic homologues are encoded by duplicated genes of the same origin: (C) histidyl-tRNA synthetase – the mitochondrial and the cytoplasmic homologues are encoded by the same gene. Names outside brackets refer to the genome encoding the protein (Mit = mitochondrial genome; Nuc = nuclear genome; Chl = chloroplast genome). Names in parentheses refer to the location of the protein (mit = mitochondria; chl = chloroplast, cyt = cytoplasm). Branch lengths are proportional to those reconstructed with the NJ method. Values at nodes indicate the percentage of 1000 NJ bootstraps, 100 MP bootstraps and 1000 ML puzzling steps, in this order ancestor of mitochondria could not have transported ATP to its host (Andersson *et al.*, 1998, Martin and Muller, 1998). This implies that the ATP/ADP translocase characteristic of mitochondria must have evolved after the intial symbiosis was established. Indeed, mitochondrial ATP/ADP translocases were earlier recognized as unrelated to those of *Rickettsia* and plastids (Andersson *et al.*, 1998; Wolf *et al.*, 1999). The present phylogenetic reconstructions (Figure 5) show that the ATP/ADP translocase lineage of mitochondria can be organized in a coherent tree that is unrelated to that generated by the bacteria/plastid lineage of translocases (Andersson, 1998; Andersson *et al.*, 1998).

Copyright © 2000 John Wiley & Sons, Ltd.

The acquisition of the ATP/ADP translocase along with components of the protein import system can be taken as a marker for the transition of the endosymbiont into an organelle.

Phylogenetic studies of the mitochondrial proteomes of other organisms will provide a more detailed description of the evolution and acquisition of the eukaryotic components of the mitochondrial proteome. Unfortunately, it seems that there is so little left of the ancestral α-proteobacterial genome in modern organisms that a description of its devolution from the mitochondrial proteome is not a forseeable project. We conclude that the evolutionary history of the mitochondrial proteome reflects the divergent histories of its two principal genomic sources: One is the reductive mode of the ancestral α-proteobacterial genome that has lost most of its genes and transferred the greater part of the remnants to the nucleus. The other is the expansive evolution of the eukaryotic nuclear genome that seems to have evolved into the major source of the mitochondrial proteome in modern organisms.

### Acknowledgements

Supported by the Swedish Natural Sciences Research Council, the Knut and Alice Wallenberg Foundation and the Swedish Foundation for Strategic Research.

### References

Altschul SF, Madden TL, Schaffer AA, Zhang J, Zhang Z, Miller W, Lipman DJ. 1997. Gapped BLAST and PSI-BLAST: a new generation of protein database search programs. *Nucleic Acids Res* **25**: 3389–3402.

Andersson SGE, Zomorodipour A, Andersson JO, *et al.* 1998. The genome sequence of *Rickettsia prowazekii* and the origin of mitochondria. *Nature* **98**: 133–140.

Andersson SGE, Kurland CG. 1999. Origin of mitochondria and hydrogenosomes. *Curr Op Microbiol* **2**: 535–541.

Andersson SGE. 1998. Bioenergetics of the obligate intracellular parasite *Rickettsia prowazekii. Biochim Biophys Acta* **1364**: 105–111.

Andersson SGE, Kurland CG. 1998. Reductive evolution of resident genomes. *Trends Microbiol* **6**: 263–268.

Burger G, Lang BF, Reith M, Gray MW. 1996. Genes encoding the same three subunits of respiratory complex II are present in the mitochondrial DNA of two phylogenetically distant eukaryotes. *Proc Natl Acad Sci U S A* **93**: 2328–2332.

Embley TM, Horner DA, Hirt RP. 1997. Anaerobic eukaryote evolution: hydrogenosomes as biochemically modified mitochondria. *Trends Ecol Evol* **12**: 437–441.

Fenchel T, Finlay BJ. 1995. *Ecology and Evolution in Anoxic Worlds*. Oxford University Press: Oxford.

Galtier NM, Gouy M, Gautier C. 1996. SeaView and Phylo_win, two graphic tools for sequence alignment and molecular phylogeny. *Comput Appl Biosc.* **12**: 543–548.

Gray MW. 1992. The endosymbiont hypothetis revisited. *Int Rev Cytol* **141**: 233–357.

Gray MW, Burger G, Lang BF. 1999. Mitochondrial evolution. *Nature* **283**: 1476–1481.

Hodges PE, McKee AHZ, Davis BP, Payne WEB, Garrels JI. 1999. Yeast Protein Database (YPD): a model for the organization and presentation of genome-wide functional data. *Nucleic Acids Res* **27**: 69–73.

Horner DS, Hirt RP, Embley TM. 1999. A single eubacterial origin of eukaryotic pyruvate–ferredoxin oxidoreductase genes: implications for the evolution of anaerobic eukaryotes. *Mol Biol Evol* **16**: 1280–1291.

Lang BF, Burger G, O'Kelly CJ, *et al.* 1997. An ancestral mitochondrial DNA resembling a eubacterial genome in miniature. *Nature* **387**: 493–497.

Lopez-Garcia P, Moreira D. 1999. Metabolic symbiosis at the origin of eukaryotes. *Trends Biotechnol* **24**: 88–93.

Martin M, Müller M. 1998. The hydrogen hypothesis for the first eukaryote. *Nature* **392**: 37–41.

Neupert W. 1997. Protein import into mitochondria. *Ann Rev Biochem* **66**: 683–717.

Olsen GJ, Woese CR, Overbeek R. 1994. The winds of (evolutionary) change: breathing new life into microbiology. *J Bacteriol* **176**: 1–6.

Saitou N, Nei M. 1987. The neighbor-joining method: a new method for reconstructing phylogenetic trees. *Mol Biol Evol* **4**: 406–425.

Schatz G. 1996. The protein import system of mitochondria. *J Biol Chem* **271**: 31763–31766.

Seoighe C, Wolfe KH. 1999. Yeast genome evolution in the post-genome era. *Curr Op Microbiol* **5**: 548–554.

Sicheritz-Ponten T, Kurland CG, Andersson SGE. 1998. A phylogenetic analysis of the cytochrome *b* and cytochrome *co* oxidase I genes support an origin of mitochondria from within the Rickettsiaceae. *Biochem Biophys Acta* **1365**: 545–551.

Strimmer K, von Haeseler A. 1996. Quartet puzzling: a quartet maximum likelihood method for reconstructing tree topologies. *Mol Biol Evol* **13**: 964–969.

Swofford DL. 1999. *Phylogenetic Analysis Using Parsimony (and Other Methods)*, Version 4. Sinauer Associates: Sunderland, MA.

Thompson JD, Higgins DG, Gibson TJ. 1994. CLUSTAL W: improving the sensitivity of progressive multiple sequence alignment through sequence weighting, position-specific gap penalities and weight matrix choice. *Nucleic Acids Res* **22**: 4683–4690.

Viale A, Arakaki AK. 1994. The chaperone connection to the origins of the eukaryotic organelles. *FEBS Lett* **341**: 146–151.

Wolf YI, Aravind L, Koonin EV. 1999. Rickettsiae and chlamydiae: evidence for horizontal transfer and gene exchange. *Trends Genet* **15**: 173–175.

Wolfe KH, Shields DC. 1997. Molecular evidence for an ancient duplication of the entire yeast genome. *Nature* **387**: 708–713.

Copyright © 2000 John Wiley & Sons, Ltd. 
